# Perceived stigma of leprosy among community members and health care providers in Lalitpur district of Nepal: A qualitative study

**DOI:** 10.1371/journal.pone.0209676

**Published:** 2018-12-27

**Authors:** Sujan Babu Marahatta, Rakchya Amatya, Srijana Adhikari, Deena Giri, Sarina Lama, Nils Kaehler, Komal Raj Rijal, Suchana Marahatta, Bipin Adhikari

**Affiliations:** 1 Manmohan Memorial Institute of Health Sciences, Swayambhu, Kathmandu, Nepal; 2 Mahidol-Oxford Tropical Medicine Research Unit (MORU), Faculty of Tropical Medicine, Mahidol University, Bangkok, Thailand; 3 Central Department of Microbiology, Tribhuvan University, Kirtipur, Kathmandu, Nepal; 4 BP Koirala Institute of Health Sciences, Dharan, Nepal; Sefako Makgatho Health Sciences University, SOUTH AFRICA

## Abstract

**Background:**

Leprosy remains a major stigmatizing condition. Stigma is a dynamic process resulting from the interaction between physical attributes caused by leprosy and the existing stereotypes in a community. Leprosy has pervasive impacts on all areas of life including psychosocial burden to an individual, social interaction, marriage, and employment. These impacts vary and are largely dependent on a particular culture and community. The main objective of this study was to explore the perceived stigma of leprosy amongst community members and health care providers in Lalitpur district of Nepal.

**Methods:**

A total of six focused group discussions (FGDs) with 43 participants from a community living close to Anandaban Leprosy Hospital and ten semi structured interviews (SSIs) with health care providers were conducted between October and December 2016. An interview guide was used for the FGDs and SSIs. All qualitative data were transcribed and translated into English and were thematically analyzed using Atlas.ti software.

**Results:**

Visible deformities due to leprosy was one of the major contributing factors for stigma. Stigma was further exacerbated by an attitude to conceal the disease due to perceived fear of potential discrimination. While over the years, stigma was felt to be decreasing, various aspects of life were still affected by leprosy stigma including marriage, employment and social interaction. This was largely attributed to leprosy and its consequences, specifically the disability and deformity caused by leprosy.

**Conclusion:**

Leprosy was still perceived to be feared and concealed because of potential discrimination, even within the community that was close to a long established leprosy hospital. Various aspects such as marriage, employment and social interaction were still affected by the stigma which was strongly associated with visible deformities. In addition to ongoing rehabilitation and stigma reduction programs, integrating strategies such as community engagement wherein community and leprosy affected person jointly take a role in stigma reduction programs can be helpful.

## Introduction

There have been great strides towards leprosy elimination with most cases now concentrated in India (60%), Brazil (13%) and Indonesia (8%) which accounted for above 80% of new cases in 2015 [1]. The World Health Organization (WHO) has set a target to interrupt the transmission of leprosy globally by 2020 [2]. Nevertheless, the paradoxical achievement of elimination may undermine the relevance of stigma and disability which continue to affect old and new leprosy affected persons [3].

Unlike other diseases with high mortality, leprosy is a slowly progressive chronic disease that can persist with the remnants of disability. Around two million people are currently living with disability related to leprosy [1], one of the main precursor of leprosy stigma [4]. Apart from the disability caused by leprosy, the perceived stigma towards leprosy affected persons has been found to be persisting in Thailand and Nepal. In Thailand, about 55% of community members perceived that staying in a community with leprosy affected persons was a shame or embarrassment [5]. In Western Nepal, 51% of community members perceived that leprosy affected persons conceal their conditions for fear of ostracization [6].

While it is completely treatable, curable, and medicines are freely available at the health centers, leprosy is still an epitome of stigma and remains to be a social disease rather than a mere biomedical term [7–10]. One of the main reasons for such a huge social burden is due to the persistence and long standing social stereotypes attached to it [5] which are often instigated and aggravated by visible attributes such as deformities, impairment and disabilities [4, 7, 11]. Visible signs of leprosy such as wet wounds, ulcers, and reactions on the skin undergoing treatment were found as stigmatizing factors in a qualitative study conducted in Eastern Nepal [12] and mixed method studies in Western Nepal [13] and Thailand [14]. These physical attributes are often interpreted and stereotyped invariably across the society, however, the nature and extent of such stigma process are unique to each culture and community.

Community perceptions and attitudes towards leprosy affected persons are a critical and unique reflector of how a society stereotypes leprosy. Community stigma towards leprosy affected persons impacts various areas of their lives such as marriage, social interaction, and employment [5, 6, 15, 16]. The main objective of this study was to explore the perceived stigma towards leprosy affected persons among the community members living close to the specialized hospital for leprosy and health care workers who provide service to leprosy patients in Lalitpur district of Central Nepal.

## Methods

### Study design and study site

This was a cross-sectional qualitative study conducted between October and December 2016 in a community living around Anandaban Leprosy Hospital, in the Southern Lalitpur district of Nepal. It is a tertiary care hospital for leprosy, operated by The Leprosy Mission (TLM), a UK based international non-government organization since 1957 [17]. Community participants were selected from the villages around the leprosy hospital.

### Participants for focused group discussions (FGDs)

In the community, Village Development Committee (VDC) representatives were first consulted and explained about the study objectives and interview procedure. The VDC representative then discussed the study with community members. A diverse range of residents were invited to participate in focused group discussions (FGDs). All invited participants were explained the study procedure and benefits by the investigators. Among the participants, a lottery method was used to select eight to ten persons for a FGD. A total of six FGDs were conducted among forty three participants.

Participants who have never heard of leprosy and have not seen a case of leprosy in the past were excluded. Willingness to participate and opting out of the study at any time without any reasons were well informed and respected. Written informed consent was obtained from the respondents before FGDs.

FGDs took place in one of the houses within the village and were conducted by an interviewer with the help of an additional note taker. All FGDs were audio-recorded in addition to the note taking.

### FGD questionnaire guide

An FGD questionnaire was adapted from the Explanatory Model Interview Catalogue (EMIC) questionnaire for leprosy [18], a reliable and validated Nepali version of which was used in the past study [6]. Themes covered in the EMIC were broadly included in an interview guide and were adapted for the FGDs. Thus developed interview guide was used for the discussion with additional probing for deeper issues related to these themes. In addition, socio-demographics of participants such as age, sex, education and VDCs were collected.

### Participants for semi-structured interviews (SSIs) and interview guide

Themes from the EMIC were further adapted to design semi-structured interviews (SSIs) for the health care providers to reflect on perceived stigma and the health seeking behavior of leprosy affected persons. All the relevant health care providers responsible for leprosy health care were contacted. An attempt was made to include staff from all levels of work experience. Ten semi-structured interviews were conducted with these health workers at their office after obtaining written informed consent.

### Sample size

The number of participants and the number of FGDs including SSIs were subject to data collection reaching theoretical saturation, whereby no novel findings emerged from subsequent interviews [19].

### Data management and analysis

All the transcriptions from interviews and focus group discussions, including the interviewer’s notes were transcribed and translated into English. All transcripts were coded line by line based on the themes of the questionnaire guide using Atlas.ti 7 (Scientific Software Development GmbH, Berlin, Germany). Data analysis employed a deductive approach based on the pre-existing themes and an inductive approach to add emerging themes [20] by two researchers who conducted the FGDs and SSIs. All researchers were invited for the discussion on finalized themes.

### Ethics approval and consent to participate

Approval for this study protocol was obtained from Institutional Review Committee (IRC), Manmohan Memorial Institute of Health Sciences. Written informed consent was obtained from each participant before conducting FGDs and SSIs.

## Results

A total of 43 participants from 6 different FGDs were included in this study (Table 1). The majority (33 out of 43) of the participants were below 45 years, and 19 were male and 24 were female. Among total participants, only 30% (13/43) could not read and write.

**Table 1:** Characteristics of participants in focused group discussions (n = 6).

Focused Group Discussions (FGDs)	Age Group		Sex		Education level		
	≤45 years	≥46 years	Male	Female	No education	Primary Education	Secondary Education
Tika Bhairav (FGD1)	6	1	4	3	1	3	3
Tika Bhairav (FGD2)	6	1	5	2	0	4	3
Tika Bhairav (FGD3)	4	3	5	2	3	4	0
Mathillo Tol (FGD4)	5	2	5	2	2	3	2
Tallo Tol (FGD5)	5	2	0	7	4	2	1
Faidol (FGD6)	7	1	0	8	3	3	2
Total	33	10	19	24	13	19	11

Recalling their past experience, community participants in the FGDs felt that perceived stigma towards leprosy was decreasing over the years and this was also echoed by health care providers during SSIs. Nevertheless, impacts of leprosy on concealing the disease, marriage, social interactions (buying food from leprosy affected persons and visiting their house) and health seeking behavior were still prevalent and are discussed below under each theme.

### General perceptions towards leprosy and affected person

Participants during FGDs asserted that the awareness level regarding leprosy has increased compared to the past. Nevertheless, participants also asserted that there was still a need for community members’ supportive attitudes towards leprosy affected persons. Many participants expressed that they did not have any negative acts towards leprosy affected persons. However, they were aware of such community discrimination and believed this to be mostly a “thing of the past”.

*Such practice of discriminating [looking down] a leprosy patient is not prevalent here or these days…….It is a thing of the past. I was admitted to a hospital some time ago and I became a friend with the leprosy patient. We used to stay together and she used to call me a sister. I used to share my food with her because she used to get food from the hospital and I used to get from my home which was tastier than hers. So, I used to share my food. She was a Tamang [Ethicity] girl. So, I do not feel any kind of hatred feeling*.

37 years, female FGD participant

Participants were vocal about how deformities and disabilities affected their attitude towards leprosy affected persons. Few participants perceived that leprosy was a highly communicable disease. Participants generally expressed that they would attempt to avoid such patients because of the fear of the transmission and status of those patients in terms of treatment completion and adherence.

*If the person’s hand and legs have already fallen off due to leprosy then we feel hesitation to go near to [approach near] them as we do not know if he/she has finished the treatment….still can transmit? We would not know and it would not matter much if the person seems physically normal*.

54 years, male FGD participant

### Concealing the disease

Participants during FGDs reported that persons affected by leprosy were reluctant to reveal their conditions to the community. Concealing the disease condition also affected their health seeking behaviour. Leprosy affected persons were perceived to disclose their condition to their close friends or family members, even though hiding the disease was thought to be more common in the past.

*There were two persons in our village who had leprosy but we were unaware about that. They used to hide the disease. They used to go to the hospital secretly and take medicines. Still at present, [these] people try others not to know about their disease [. . . . . . . . .] Since the hospital is near, so, some people go there secretly and take medicine while some even stay [at home] by suppressing the disease*.

57 years, female FGD participant

### Visiting leprosy affected person’s house

Participants were asked about how they felt while visiting the house of a leprosy affected person. Participants at FGDs shared that they would feel uneasy visiting the house of leprosy affected persons if they had visible deformities and disabilities. Some were reluctant to visit because of the fear of transmission. While others knew transmission was unlikely, they were still hesitant to visit due to community’s negative perceptions towards leprosy.

*We feel somewhat uncomfortable if that person has lost his fingers… Anyway, I have eaten food prepared by her [Leprosy affected person] about three times…. Though I know that the disease is not communicable but still I felt a kind of uneasy*.

27 years, female FGD participant

### Buying foods from leprosy affected persons

Unease and hesitation surrounding buying food from leprosy affected persons were reiterated in all FGDs. Hesitation about buying food from leprosy affected persons was often linked to fear of potential transmission. Buying food from leprosy affected persons was often avoided because community members did not prefer their food to be handled by leprosy affected persons. Some community members were less likely to visit the shops owned by leprosy affected persons.

*We prefer buying food from some other people than leprosy affected persons. If a leprosy affected person is there in the shop then we prefer going later when another person will replace him/her*.

44 years, female FGD participant

### Employment of leprosy affected persons

Participants perceived that leprosy affected persons would not have any problems in getting employment if they were skilled, qualified and physically capable of performing the job. However, some participants clearly mentioned that leprosy affected persons would not be given a job if he or she has a physical deformity. Employment was not just rooted in the capacity to execute the work, but to the broader ramifications of community stigma, such as how customers would potentially be deterred to visit their place.

*If they [leprosy affected persons] have qualifications then they will definitely get the job. But if they have physical limitations or are handicapped…. finding a job may be problematic. Sometimes, they will not be preferred based on the visible impairments, considering how customers would feel about it [for example in restaurant], even if they can perform the given job*.

31 years, female FGD participant

### Leprosy and marriage

*Impacts of leprosy on marriage of the leprosy affected person or their relatives were rooted in the fear of potential disease transmission*.

*Nobody marries a person with leprosy. Who will knowingly marry a person with leprosy? People do not marry considering that the disease may transmit to them. However, a leprosy affected person does marry another leprosy affected person [thinking for a while]…….but a person with leprosy and a person without leprosy do not marry each other. I have not seen such things happening*.

31 years, female FGD participant

Participants expressed that there were no problems for a marriage between two leprosy affected persons. However, physical and visible deformities were recognized to affect the marriage of all alike. Others opined that it was dependent on a partner’s choice and ultimate decision would depend upon the marrying partners. Upon further probing, community members expressed that impacts on marriage were largely due to social stigma—*“how society thinks when somebody has brought a leprosy affected person at home”*. On the more personal level, there were concerns of the community stigma to physical deformities of the spouse, and the responses to community inquiries they would have to deal with.

### Difficulties and discrimination for a family of leprosy patient

While participants expressed that availability of treatment has greatly palliated the burden of leprosy, the historical association of leprosy as a derogatory term was deeply entrenched in the Nepalese culture. Family members of leprosy affected persons may experience community discrimination.

*……Villagers might say, “Your family member has this disease*.*”…..They might say, “Offspring of such type of people [“esta ko santaan”]-a derogatory term (that literally denounces as a sibling of ostracized class)………*

44 years, female FGD participant

### Opinions of health service providers towards perceived stigma of leprosy and impact on health seeking behaviour

The majority of the health service providers, during semi-structured interviews (SSIs), expressed that there was a high level of stigma related to leprosy in the past. Historically, leprosy was associated with “curse” and “uncleansed spirits” which resulted in “discrimination, isolation and loathe”. SSI participants echoed that the level of stigma was decreasing steadily over the years.

During SSIs with a hospital training coordinator and a heath assistant, it was clearly stated that community stigma was strongly associated with disability and deformities. A health service provider stated *“Leprosy patients are discriminated because of their physical deformities*. *They lose their fingers*, *toes and even the whole organ or body parts*.*”*

Health care providers were concerned that leprosy affected persons were generally seeking traditional health practitioners instead of seeking treatment at the leprosy hospital. Attending traditional health practitioners may delay treatment of leprosy affected persons, further worsening their condition. Further explanations on the health consequences were spontaneously mentioned and included *“the anesthesia of the lesion and the tendency to un-notice the lesion”*. Such an anesthetic property of the lesion was heavily attributed to the *“delayed health seeking behavior*.*”* In addition, such delayed health seeking behavior were attributed to *“development of deformities”* and the subsequent stigma. Leprosy affected persons were also attending spiritual healers [*Dhami Jhakri*] in addition to a standard treatment at the hospital.

Health care practitioners also directly observed differences in societal perception with older generations harboring more stigma.

*When I worked in rural area, I found [saw] an elderly leprosy patient who was under the treatment and was receiving medicines. I found [saw] a young leprosy patient as well. I did not find any kind of stigma among young generations. In case of elderly people, they had some kind of stigma regarding leprosy. They had a belief that leprosy was caused due to wrath of god and goddess. As the complication of leprosy leads to disfiguration of the affected organ, the leprosy affected person felt uncomfortable to stay with their family and get involved in the social activities. They had lots of restrictions in the society because of which they did not want to come out in the society. They had to live an isolated life*.

27 Years old male medical officer

## Discussion

This is the first qualitative study amongst community members and health care providers living in a community with a hospital specialized for leprosy in Central Nepal. While both community members and leprosy health care workers agreed that level of stigma was decreasing, both groups believed that it was still possible and was associated with visible physical deformities and societal stereotypes and affected many aspects of leprosy affected persons’ lives.

Association of leprosy stigma with visible deformities and stereotypes found in this study is consistent with previous studies conducted in both Western [6] and Eastern [15] Nepal, and is a global phenomenon [9, 21]. These associations have been globally prevalent and apply to many other stigmatizing conditions including leprosy [8, 21–25]. Consistent with Nepalese communities, in Thailand, community members still harbored such beliefs and were likely to have more stigma prompted by visible deformities [5].

This study found that persons affected with leprosy would as much as possible attempt to conceal the disease. In addition, if they had to, leprosy affected persons would rather distort the truth and would mention it as some other disease. This clearly reflects how much community stigma is rooted to the leprosy terminology itself. The fear of being discriminated and isolated from the community prompts leprosy affected persons to hide the disease from the community. This has been consistent with studies conducted in Western Nepal where concealing the condition was the most affected aspect of stigma in leprosy [6, 13, 26]. While concealing the disease condition itself has been relatively well recognized, the wider implications such as delayed treatment and consequent disability development have grave consequences [4, 11, 27]. As an example, adolescents in India were hiding the disease and delaying the treatment because of social stigma and the fear of potential discrimination [28].

Willingness to visit a leprosy affected person’s house was heavily interlinked with the visible attributes of leprosy, such as deformities and disabilities. This further attests the previous evidence that disability and deformities, specifically if they are visible, are likely to increase the level of stigma and decrease the extent of social interaction [6, 9, 11, 13, 29, 30].

Disabilities in general contribute to the development of community stigma, however, disabilities related to leprosy exacerbates the level of stigma [29]. In addition, there are various other social factors that contribute to higher stigma restricting social interaction. For instance, females affected by leprosy develop more stigma when compared to males [31, 32] and various other factors such as ethnicity, caste and socio-economic status can equally contribute to social stigma and interaction [6, 9, 13]. Thus it further explains how multitude of social and contextual factors together with the disease itself are responsible for a social construct of stigma.

Relatively few community participants expressed hesitation to buy food from leprosy affected persons. The fear of disease transmission and the appearance of the visible deformities and wounds were the main reasons for avoiding buying food from leprosy affected persons [5, 6, 13, 26]. While perceived biomedical reasons such as fear of transmission was found heavily prevalent in Western Nepal as well [13], the tendency to avoid them if possible cannot be explained entirely by “fear of transmission”. The social construction of fear embedded in stereotypes that leprosy is caused by “sin”, “bad deeds in the past” and is “dirty” or “uncleansed spirits” further explains the avoidance of buying and exchanging foods or goods from leprosy affected persons [15, 26].

Impacts of leprosy on marriage were perceived to be decreasing over the years. However, recent studies from the eastern region of Nepal have shown the pervasive impacts of leprosy on marriage such as; cancellation of marriage after knowing the leprosy status, difficulty finding partners, divorce, sexual abuse, and domestic violence [33].

In this study, both community members and health care providers expressed the impacts of physical appearance and the deformities as the main factors affecting marriage. While it is a global phenomenon that marriages are affected by physical appearance and thus deformities and disability, in Nepal marriages are influenced by various other social factors that include, caste, ethnicity and social status. In light of these contextual and cultural factors that affect marriage, a family member associated with a leprosy affected person or a leprosy affected person can have added difficulties in finding a partner and thus marriage [32, 33]. In some cases, leprosy affected persons sharing leprosy as a common social phenomenon have been able to marry each other either through a relative’s recommendation or when they have met each other, for example in the hospital [26]. However, in Eastern Nepal the power differentials between a husband and a wife, even if both of them are affected by leprosy, prevailed to an extent that the man had extra leverage to go for a second marriage, withholding of sex and domestic violence [33].

In contrast to our study area, multiple factors were found to affect marriage in South-eastern Nepal where a strong culture of dowry and patriarchy is prevalent [34]. In these communities, leprosy affected women were vulnerable to heavy household workload, sexual abuse and domestic violence which can further exacerbate the impacts of leprosy on marriage.

Employment of leprosy affected persons was generally correlated with the skill, education level, and absence of deformities. These findings were consistent with earlier studies conducted in various parts of Nepal [6, 15, 16]. Inability to perform activities can disqualify a leprosy affected person from employment, however, exclusion from the job based on the deformities alone was common in South-eastern Nepal and this may be common in other regions of Nepal [16]. Furthermore, the embedded social fear of transmission associated with the wound and the deformities could be another reason for exclusion from the employment.

### Community engagement as a potential stigma reduction strategy

Building on the conceptual framework of stigma previously outlined, stigma are categorized into six various types [27, 35]. Three are from perpetrators and three from those who are stigmatized. Stigma from perpetrators result in accepted, endorsed and enacted stigma (acts of discrimination). Stigmatized individual can evince anticipated (or perceived), internalized (or self- stigma) and enacted (or experienced) stigma [35, 36]. Community stigma towards leprosy are still prevalent and the extent and characteristics of such stigma are largely dependent on the local social and cultural context [6]. While various stigma reduction strategies such as public contact and rights based counselling interventions have shown encouraging results [37, 38], these could be augmented by a more pragmatic approach using elements inherent in community engagement [39, 40]. The elemental framework of community engagement has been successfully implemented for control and elimination of other diseases [41–43] and can be adapted to design stigma reduction strategies for leprosy (Table 2).

**Table 2:** Elements of community engagement [40] and the possible methods of its use in stigma reduction strategies.

Elements of community engagement	Description	Illustration
Stakeholder and authority engagement	A stepwise approach of engaging authorities and stakeholders at various levels for a community wide interventions can be the first step.	Authority and stakeholders working in the field of leprosy stigma are consulted, to develop a community wide approach for a stigma reduction strategy.
Local human resources	In the targeted communities, selecting, training and devolving community members with the stigma reduction strategies can be a second step. Local human resources can best represent the community and therefore, their role can be pivotal in rolling out the intervention.	Both leprosy affected persons and the representative of targeted community can be selected, trained and provided with the responsibility for a stigma reduction campaign.
Formative research	Formative research in the targeted communities can provide important insights on designing and executing a tailored intervention. This may include qualitative and quantitative studies, observations and meeting with the local community members to explore the local social and cultural context.	In the targeted communities, through formative research, the rich details on the process of stigma, cultural entanglements, and the way to counteract can be explored and thus can become pivotal in further refining the tailored interventions. The findings from this current study can be used as an initiation of formative research.
Responsiveness	Despite a design of a tailored intervention, a responsive approach is critical to address the needs and issues of the community. This also requires a flexibility in approach to adapt the needs of the community.	In contrast to a fixed set of strategies to counteract the stigma, a responsive approach involves a flexibility in adaptation of the strategies, that for example includes reviewing and readjusting the intervention for a particular community, a cohort or the members of the community (both affected and unaffected by leprosy).
Sharing control and leadership with the community	In contrast to a vertical approach, sharing control and leadership with the community members (perpetrators) and the leprosy affected persons (stigmatized persons) can provide a joint platform to execute and design an intervention for a particular community.	Executing a stigma reduction strategy together with the community members and leprosy affected persons in a particular community can render a sense of ownership and responsibility for its implementation. In addition, community led implementation can boost more trust in the community and may become more sustainable than a vertically implemented intervention.

In contrast to a vertically instituted leprosy stigma reduction program, community engagement can encourage community members together with the leprosy affected persons to jointly design a locally tailored intervention for a particular community. This has been the case in recent targeted malaria elimination studies in the Greater Mekong Sub-region where community members together with the concerned authorities piloted a malaria elimination project [40, 44–48]. Such a process of engagement also engenders trust together with a sense of ownership and responsibility to the community and may become more economic and sustainable than vertically implemented programs which are often constrained by budget and time [40, 43]. The findings from this study could be used to guide future community engagement strategies to counteract leprosy stigma.

### Strengths and limitations

This study incorporated FGDs amongst community members living close to Anandaban Leprosy Hospital and SSIs with the health care providers for leprosy. However, since this study was conducted amongst community members living in a relatively urban region within Kathmandu valley, it may not reflect the true stigma present in rural regions of Nepal or elsewhere. There could have been a high social desirability bias to answer questions in such a way to demonstrate less stigma towards leprosy. Attempts were made with respondents to reflect on “how community reacts” than on how they would as individuals to minimize biases. Future studies should also explore the perceived stigma amongst the leprosy affected persons.

## Conclusion

Leprosy was still perceived to be feared and concealed because of potential discrimination, even within the communities that were close to a long established leprosy hospital. Various aspects such as marriage, job, and food sharing were still affected and the stigma was strongly associated with visible deformities. Community engagement could be utilized to enhance the ongoing stigma reduction strategies such as health education, rehabilitation, public contact and rights based counselling interventions. Community engagement can provide community members and leprosy affected persons a joint platform to take a lead in delivering a community based health education in addition to increasing social participation and interaction.
